# Supplementary material for: Optineurin links Hace1-dependent Rac ubiquitylation to integrin-mediated mechanotransduction to control bacterial invasion and cell division
Source: Nat Commun. 2022 Oct 13;13:6059. doi: 10.1038/s41467-022-33803-x (PMC9561704; doi:10.1038/s41467-022-33803-x)
Supplement: Supplementary file 1 — Supplementary Information [file 41467_2022_33803_MOESM1_ESM.pdf]

Supplementary information:

Optineurin links Hace1-dependent Rac ubiquitylation to integrin-mediated mechanotransduction to control bacterial invasion and cell division

Serena Petracchini[1, #], Daniel Hamaoui[2, 3, #], Anne Doye[2, 3], Atef Asnacios[4], Florian Fage[4], Elisa Vitiello[5], Martial Balland[5], Sebastien Janel[6], Frank Lafont[6], Mukund Gupta[7], Benoit Ladoux[7], Jerôme Gilleron[8], Teresa M. Maia[9, 10, 11], Francis Impens[9, 10, 11], Laurent Gagnoux-Palacios[12], Mads Daugaard[13, 14], Poul H. Sorensen[15], Emmanuel Lemichez[1, 2, 3*] and Amel Mettouchi[1, 2, 3*]

1 Institut Pasteur, Université Paris Cité, CNRS UMR6047, INSERM U 1306, Unité des Toxines Bactériennes, F-75015 Paris, France.

2 Université Côte d'Azur, INSERM, C3M, Team Microbial toxins in host-pathogen interactions, Nice, France

3 Equipe labellisée La Ligue contre le Cancer

4 Université Paris Cité, CNRS, Laboratoire Matière et Systèmes Complexes, UMR7057, F-75013 Paris, France.

5 Université Grenoble Alpes, CNRS, LiPhy, F-38000 Grenoble, France

6 Université de Lille, CNRS, INSERM, CHU Lille, Institut Pasteur de Lille, U1019-UMR9017, CIIL- Center for Infection and Immunity of Lille, F-59000 Lille, France

7 Université Paris Cité, CNRS, Institut Jacques Monod, F-75013 Paris, France

8 Université Côte d'Azur, INSERM, C3M, Team Cellular and Molecular Pathophysiology of Obesity and Diabetes, Nice, France.

9 VIB-UGent Center for Medical Biotechnology, VIB, Ghent, Belgium

10 Department of Biomolecular Medicine, Ghent University, Ghent, Belgium.

11 VIB Proteomics Core, VIB, Ghent, Belgium.

12 Université Côte d'Azur, CNRS, INSERM, Institut de Biologie Valrose (iBV), Nice 06108, France

13 Vancouver Prostate Centre, Vancouver, BC, V6H 3Z6, Canada.

14 Department of Urologic Sciences, University of British Columbia, Vancouver, BC, Canada.

15 Department of Molecular Oncology, BC Cancer Research Center, University of British Columbia, Vancouver, BC V5Z1L3, Canada.

# These authors contributed equally

* These authors jointly supervised this work

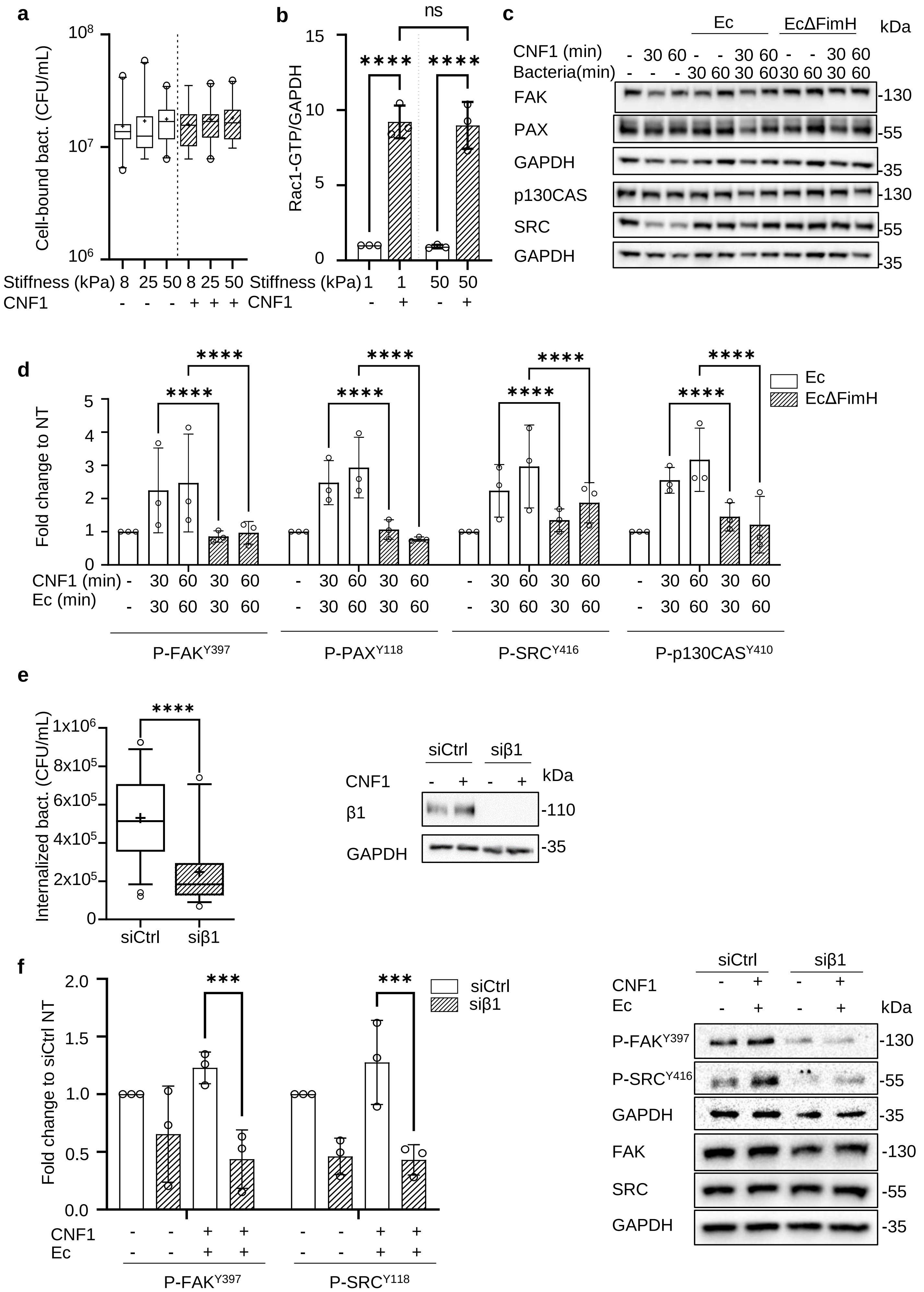
a
Cell-bound bact. (CFU/mL)
Stiffness (kPa) 8 25 50 8 25 50
CNF1 - - - + + +
b
Rac1-GTP/GAPDH
ns
****
Stiffness (kPa) 1 1 50 50
CNF1 - + - +
c
Ec
EcΔFimH
kDa
CNF1 (min)
Bacteria(min)
FAK
PAX
GAPDH
p130CAS
SRC
d
Fold change to NT
Ec
EcΔFimH
CNF1 (min)
Ec (min)
P-FAKY397
P-PAXY118
P-SRCY416
P-p130CASY410
e
Internalized bact. (CFU/mL)
siCtrl
siβ1
CNF1
β1
GAPDH
f
Fold change to siCtrl NT
***
CNF1
Ec
P-FAKY397
P-SRCY118
P-FAKY397
P-SRCY416
GAPDH
FAK
SRC

Supplementary FIGURE 1 (a-d)

a) Quantification of Ec bound to cells cultured on ECM of increasing stiffness (8,25,50kPa). CNF1 was added at 1nM where indicated(+). Boxplots displays absolute number of CFU/ml (Yaxis starts at 10^6)of n=3independent experiments and at least 3 replicates/condition, MOI100. One-way Anova with Dunnett's test for multiple comparison:not significant. **b)** Quantification of active Rac1 levels(Rac1-GTP) upon CNF1 treatment of HUVEC cells cultured on ECM of 1 and 50kPa stiffnesses, measured by effector affinity purification followed by immunoblotting. The relative densitometric analysis of Rac1-GTP over GAPDH signals was set to 1 for non-treated cells cultured on 1kPa ECM. Bars represent means±SD, n=3 independent experiments. Two-way Anova with Sidak's test for multiple comparisons : ns, not significant,****, p≤0.0001.**c)**Representative immunoblots for total levels of FAK,PAX,SRC,p130Cas corresponding to the experiment shown in Figure1b. GAPDH was used as a loading control. **d)**Quantification of phospho-protein levels for phospho-FAK(tyr-397), phospho-Paxillin(tyr-118), phospho-Src(tyr-416) and phospho-p130CAS(tyr-410) in HUVEC infected with Ec or EcΔFimH, in presence of 1nM CNF1 (level of phospho-proteins relative to GAPDH and normalized to the non-treated condition). Bars represent means±SD of n=3 independent experiments(single dots). Two-way Anova with Tukey's test for multiple comparisons:****, p≤0.0001. Expression of FAK,PAX,Src and p130CAS are not modified in our experimental conditions. **e)On the left:** Quantification of internalized Ec in HUVEC knocked down for beta1integrin(siβ1) or control(siCTRL) after30minutes of gentamicin treatment in presence of 1nM CNF1(Boxplot). Boxplots Absolute number of CFU/mL,MOI100of n=3 independent experiment and 3replicates per condition. Wilcoxon rank sum test (two-tailed):****,p≤0.0001. **On the right:**One representative immunoblot for knock-down verification of beta1integrin(siβ1), (GAPDH:loading control). **f)Left:** Immunoblot quantification of phosphorylated FAK(tyr-397) and SRC(tyr-416) in siCTRL(white) and siβ1(hatched) transfected cells, either non-treated(-)of upon Ec infection in presence of 1nM CNF1 for 30 minutes(+)(densitometric analysis of phospho-proteins relative to GAPDH, normalized to siCTRL non-treated condition). Expression of total level of FAK and Src are not modified in our experimental conditions. Bars represent mean±SD of n=3 independent experiments(single dots). Two-way Anova with Tukey's test for multiple comparisons:***,p=0.0004.**Right:**Representative immunoblot showing phosphorylated FAK and SRC in siCTRL and siβ1 HUVEC cells, either non-treated(-)of upon Ec infection in presence of 1 nM CNF1 for 30minutes(+).

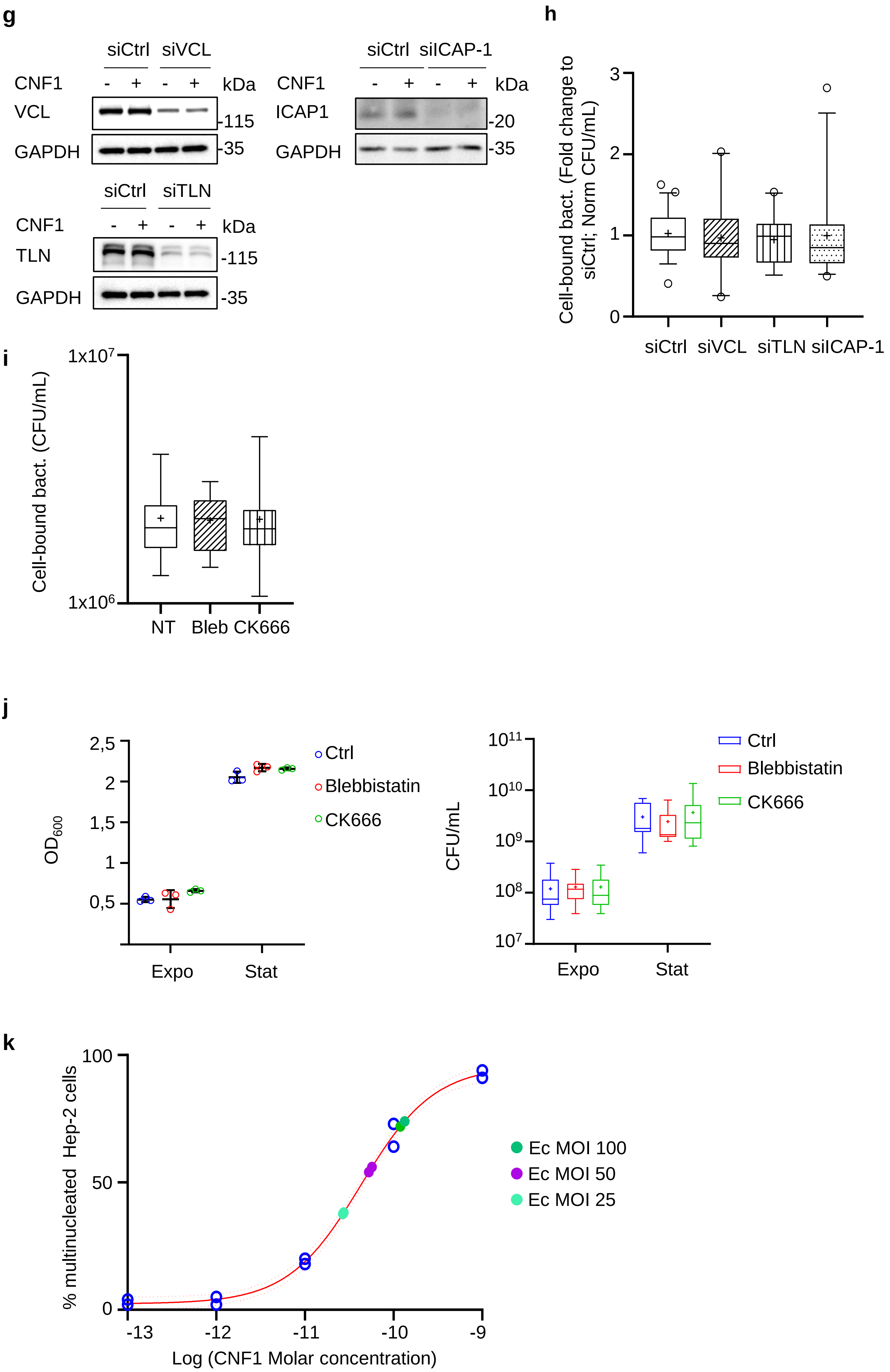
g
siCtrl
siVCL
CNF1
-
+
kDa
VCL
GAPDH
-115
-35
siCtrl
siICAP-1
ICAP1
-20
-35
siCtrl
siTLN
TLN
-115
-35
h
Cell-bound bact. (Fold change to siCtrl; Norm CFU/mL)
0
1
2
3
siCtrl
siVCL
siTLN
siICAP-1
i
Cell-bound bact. (CFU/mL)
1x10^7
1x10^6
NT
Bleb
CK666
j
OD600
2,5
2
1,5
1
0,5
Expo
Stat
Ctrl
Blebbistatin
CK666
CFU/mL
10^11
10^10
10^9
10^8
10^7
k
% multinucleated Hep-2 cells
100
50
0
-13
-12
-11
-10
-9
Log (CNF1 Molar concentration)
Ec MOI 100
Ec MOI 50
Ec MOI 25

Supplementary FIGURE 1 (g-k)

g) Representative Immunoblots showing knock down validation of Talin(TLN), Vinculin(VCL) and ICAP-1 in experiments quantified in Figure1d. GAPDH was used as loading control.

h) Quantification of cell-bound Ec to siCTRL, siVCL, siTLN or siICAP1 transfected HUVEC cells in presence of 1nM CNF1 at 30min post-infection. Boxplots displays the quantification of CFU/ml relative to the control condition (siCTRL)of n=3 (TLN,VCL) or n=5 (SiICAP1)independent experiments, 3 replicates per condition. One-way Anova:not significant.

i) Quantification of Ec bound to HUVEC cells either non treated or treated with blebbistatin(20 mM) or CK666(250mM) in presence of 1nM CNF1. Boxplots displays absolute number of CFU/mL, n=2 independent experiments, with 6 replicates per condition, MOI100. One-way Anova with Dunnett's correction test for multiple comparisons: not significant

j) Left: Optical densities(600nm wavelength) at Exponential(Expo)and Stationary(Stat)phases of Ec grown in presence of blebbistatin(20 mM) or CK666(250mM). Bars represent mean±SD of one representative experiment with 3 biological replicates(single dots). Two-way Anova with Dunnet's correction for multiple comparisons: not significant. Optical density is not significantly modified by blebbistatin and CK666 chemical inhibitors. **Right:** Boxplots representing absolute number of CFU/mL at Exponential(Expo)and Stationary(Stat)phases for Ec grown in presence of blebbistatin(20 mM) or CK666(250mM) (one representative experiment with 3 biological replicates). Two-way Anova with Dunnett's test for multiple comparisons: not significant.

k) Quantification of CNF1 production by an Ec culture equivalent of MOI100, using the Hep-2 cells multinucleation cytotoxicity assay. A standard curve was generated from the treatment by recombinant CNF1 toxin(open blue dots), and used to interpolate the molar concentration produced by 2.5,1.25 and 0.625x10^7 bacteria(corresponding to MOI100,50 and 25 respectively) using PRISM (Sigmoidal, 4PL, X is log(concentration). One experiment with 3independent conditions and 2replicates per condition. Ec used for infection at MOI100 produce a mean value of 0.11nM±0.01 of CNF1.

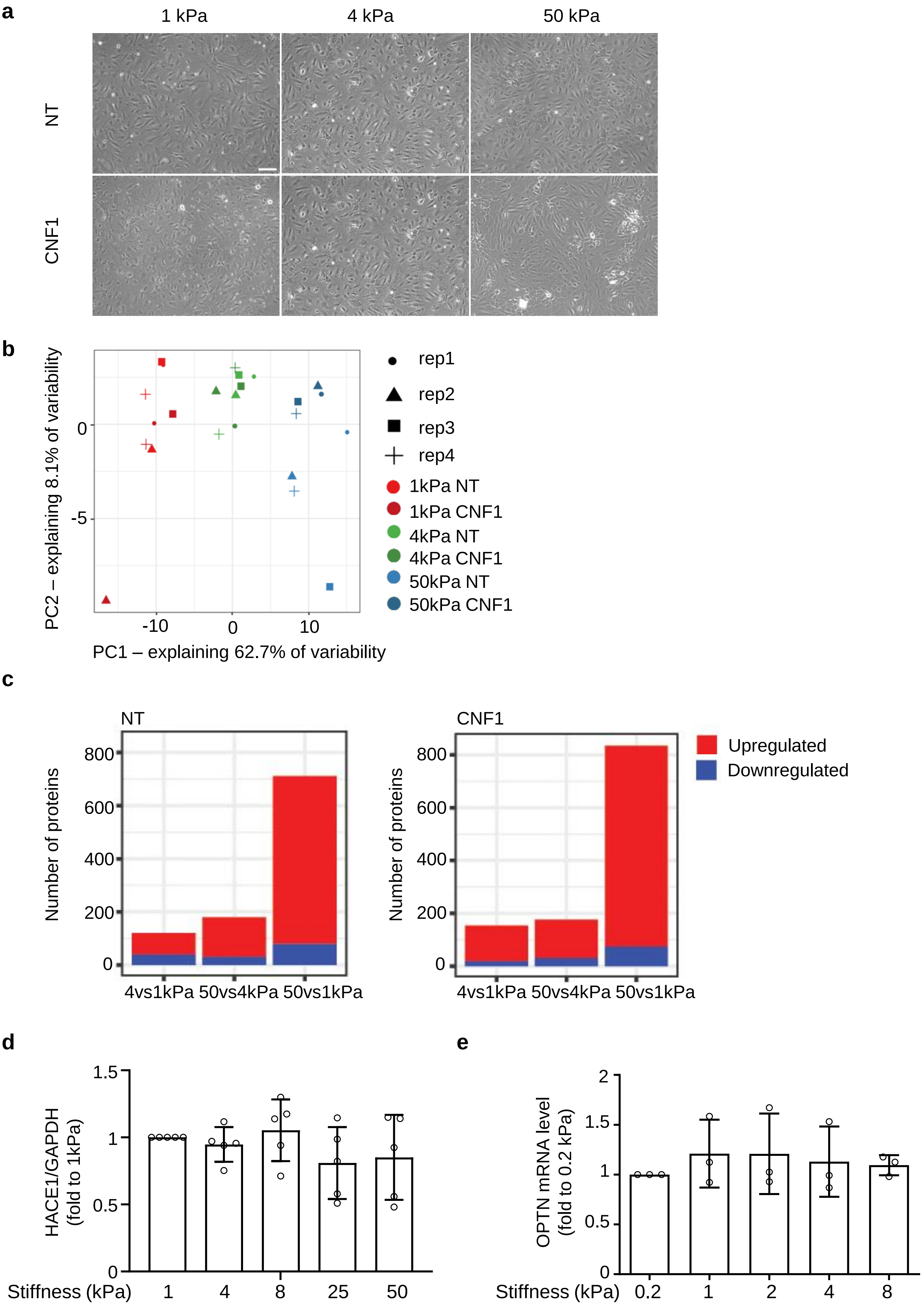

a
1 kPa
4 kPa
50 kPa
NT
CNF1
b
PC2 – explaining 8.1% of variability
PC1 – explaining 62.7% of variability
0
-5
-10
0
10
rep1
rep2
rep3
rep4
1kPa NT
1kPa CNF1
4kPa NT
4kPa CNF1
50kPa NT
50kPa CNF1
c
NT
CNF1
Number of proteins
800
600
400
200
0
4vs1kPa 50vs4kPa 50vs1kPa
Upregulated
Downregulated
d
HACE1/GAPDH (fold to 1kPa)
1.5
1
0.5
0
Stiffness (kPa) 1 4 8 25 50
e
OPTN mRNA level (fold to 0.2 kPa)
2
1.5
1
0.5
0
Stiffness (kPa) 0.2 1 2 4 8

Supplementary FIGURE 2

a) HUVEC cells plated overnight on fibronectin ECM of different stiffnesses (1-4-50 kPa) and either non treated (NT) or intoxicated with CNF1 for 2h. Phase contrast microscopy, scale bar= 100µm.

b) Principal component analysis visualization of the four replicates per condition from the proteomics experiment in Figure 2 a-c. Different stiffnesses are represented by different colours, each replicate by a different dot shape. X axis: PC1, Y axis, PC2.**c)** Barplots of significantly upregulated and downregulated proteins from the proteomics experiment in Figure 2 a-c, grouped for non treated (NT) and CNF1 treated conditions (CNF1). Y axis represents the number of proteins in the defined comparisons. A detailed description of the analysis can be found in the methods section.

d) Quantification of HACE1 protein level from western blot analysis by densitometry, cells treated as in Figure 1d. GAPDH was used as loading control. The HACE1 signal was normalized to that of GAPDH and expressed as fold relative to the signal on 1kPa ECM, set to 1. Bars represent means ± SD of n=5 independent experiments (single dots). One-way Anova with Dunnett's test for multiple comparisons: not significant.

e) Quantification of Optineurin (OPTN) mRNA levels in HUVEC cells cultured 15 hours on fibronectin-coated hydrogels of the indicated stiffness (kPa), where protein levels are linearly increasing. Bars represent means ± SD of n=3 independent experiments (single dots). One-way Anova: not significant.

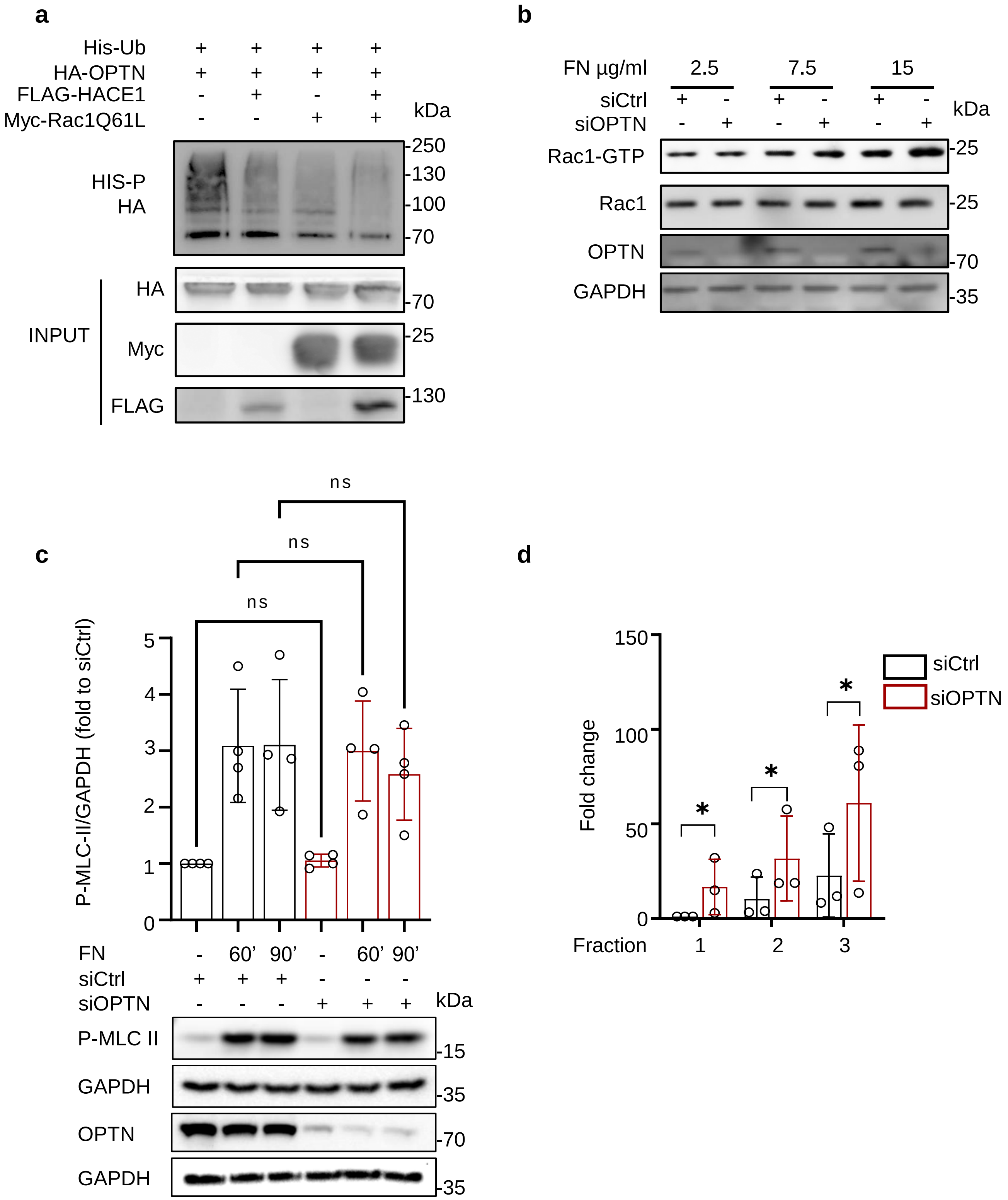
a
His-Ub
HA-OPTN
FLAG-HACE1
Myc-Rac1Q61L
kDa
HIS-P
HA
250
130
100
70
INPUT
HA
70
Myc
25
FLAG
130
b
FN µg/ml
2.5
7.5
15
siCtrl
siOPTN
kDa
Rac1-GTP
25
Rac1
25
OPTN
70
GAPDH
35
c
ns
ns
ns
P-MLC-II/GAPDH (fold to siCtrl)
0
1
2
3
4
5
FN
60'
90'
60'
90'
siCtrl
siOPTN
kDa
P-MLC II
15
GAPDH
35
OPTN
70
GAPDH
35
d
150
100
50
0
Fold change
siCtrl
siOPTN
*
*
*
Fraction
1
2
3

Supplementary FIGURE 3

a) Immunoblots showing OPTN ubiquitylation profile. Cells were transfected with expression vectors for Histidine-tagged ubiquitin, HA-OPTN and Flag-HACE1 when indicated, with or without Myc-Rac1Q61L. Covalently bound His-ubiquitylated proteins were purified (HIS-P) and resolved on 12% SDS-PAGE before anti-HA immunoblotting to reveal ubiquitylated OPTN. Input corresponds to immunoblots on 0.5% total lysate to control protein expression levels. Representative experiment, n=2.

b) Immunoblot showing levels of active Rac1 (Rac1-GTP) in HUVECs transfected with siCtrl or siOPTN and stimulated by 1h adhesion on fibronectin-coated plates at different concentrations (FN, 2.5, 7.5 and 15 µg/ml) in defined medium. Levels of Rac1, OPTN and GAPDH for loading control are assessed on 2% total lysates. Representative experiment, n=3.

c) Quantification of Myosin light chain-II phosphorylation (P-MLC II) in control (siCtrl) or Optineurin knocked down (siOPTN) HUVEC cells, detached (FN "-") and adhered for 60 and 90 minutes on 15µg/mL fibronectin-coated plates. GAPDH was used as loading control, data show fold changes compared to detached siCtrl cells. Bars represent means ± SD from n=4 independent experiments (single dots). One-way Anova with Tukey's test for multiple comparisons, ns: not significant. Immunoblots below show P-MLC-II, OPTN and GAPDH protein levels in one representative experiment.

d) Quantification of Rac1 levels in detergent resistant membrane fractions 1, 2 and 3 as in Figure 2d. Rac1 signal was first normalized to total lysate 2% (Tot). Bars represent means ± SD of n=3 independent experiments (single dots). Two-way Anova with Sidak's correction test for multiple comparisons: *, p=0.0368.

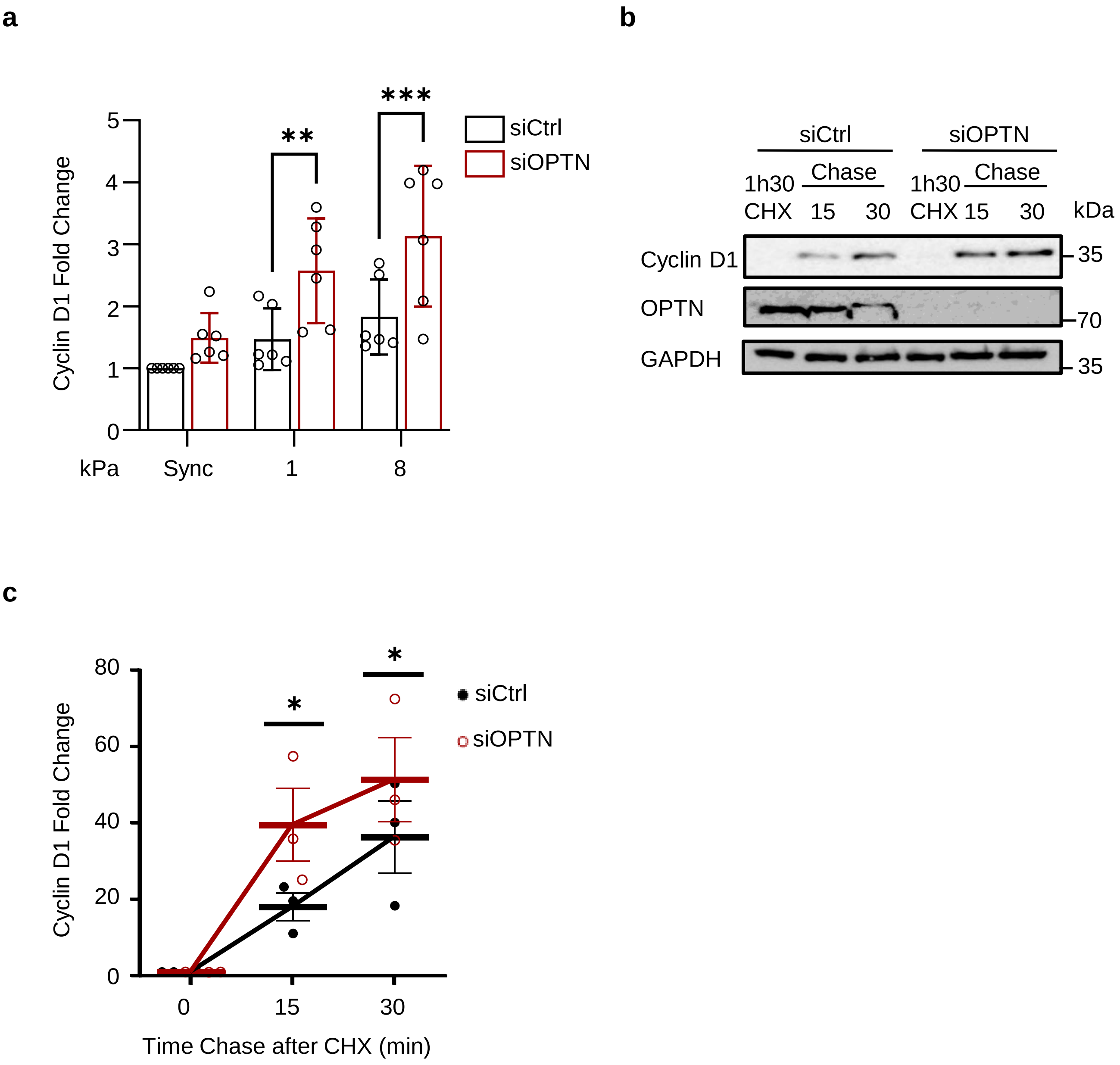
a
Cyclin D1 Fold Change
0
1
2
3
4
5
**
***
siCtrl
siOPTN
kPa
Sync
1
8
b
siCtrl
siOPTN
1h30
CHX
Chase
15
30
1h30
CHX
Chase
15
30
kDa
Cyclin D1
OPTN
GAPDH
35
70
35
c
Cyclin D1 Fold Change
0
20
40
60
80
*
*
siCtrl
siOPTN
0
15
30
Time Chase after CHX (min)

Supplementary FIGURE 4

a) Quantification of cyclin D1 protein level in cells treated as in Figure 6b. Densitometry was normalized to value in synchronized siCtrl cells, set to 1. GAPDH was used as loading control. Bars represent means ± SD of n=6 independent experiments (single points). Two-way Anova with Sidak's correction test for multiple comparisons: **, p=0.0013, ***, p=0.0004.

b) Immunoblots showing the rate of cyclin D1 expression after 15 and 30 minutes of release from protein synthesis blockage with 10 nM Cycloheximide (CHX). Immunoblots anti-OPTN and GAPDH show controls of OPTN knockdown and protein loading, respectively. Representative experiment, n=3.

c) Quantification of cyclin D1 neo synthesis in cells treated as in Supplementary Figure 4b. Densitometry was normalized to value in synchronized siCtrl cells, set to 1. GAPDH was used as loading control. The graph represent means ± SD from n=3 independent experiments (single dots). Two-way Anova with Sidak's test for multiple comparisons: 15 min *, p=0.0143; 30min *, p=0.0489.

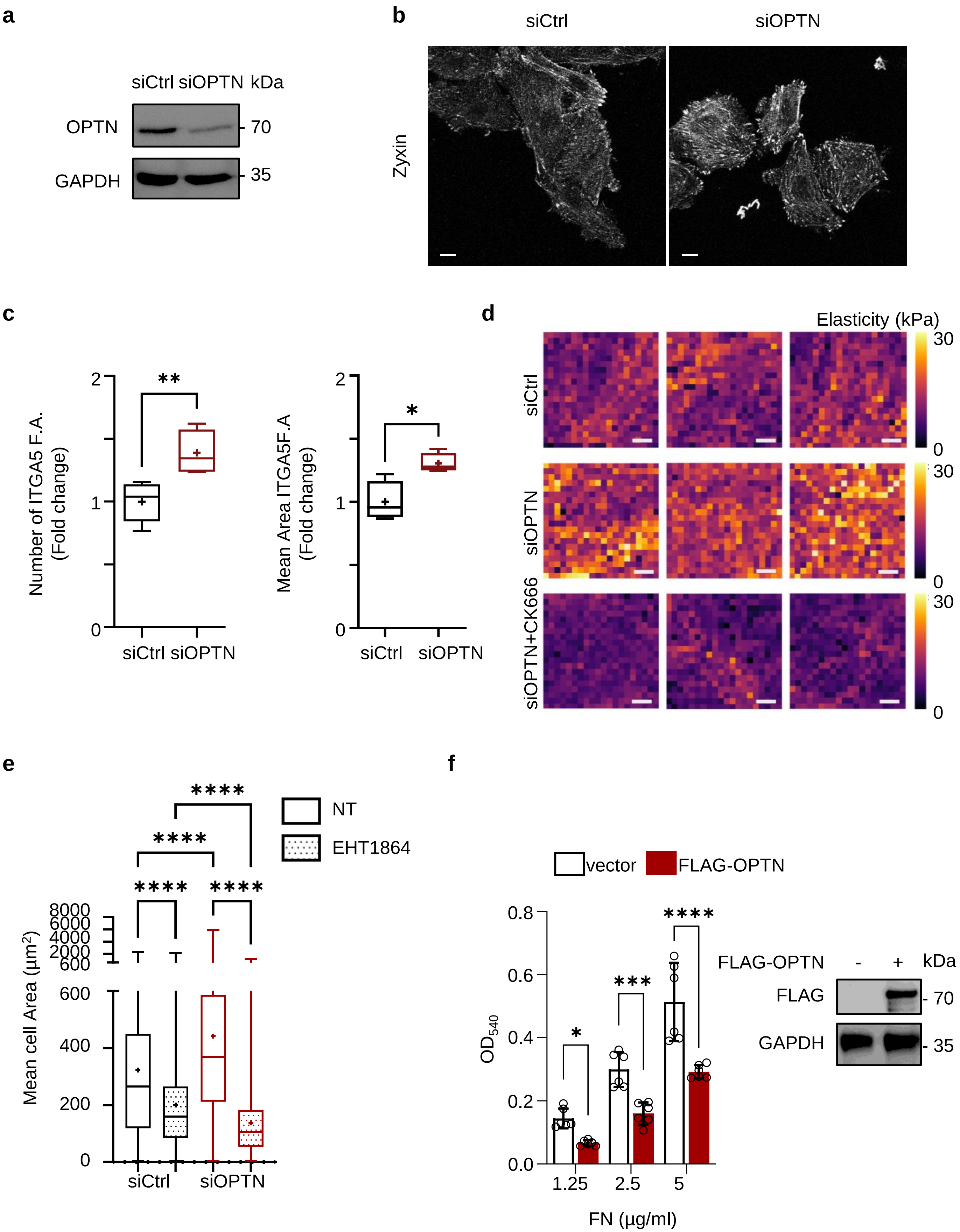

a
siCtrl siOPTN kDa
OPTN
70
GAPDH
35
b
siCtrl
siOPTN
Zyxin
c
Number of ITGA5 F.A. (Fold change)
Mean Area ITGA5F.A (Fold change)
**
*
siCtrl siOPTN
d
Elasticity (kPa)
siCtrl
siOPTN
siOPTN+CK666
30
0
e
NT
EHT1864
****
Mean cell Area (µm²)
siCtrl
siOPTN
f
vector
FLAG-OPTN
OD540
FN (µg/ml)
1.25
2.5
5
*
***
****
FLAG-OPTN - + kDa
FLAG
GAPDH
70
35

Supplementary FIGURE 5 (a-f)

a) Immunoblots showing OPTN depletion in transfected cells shown in Figure5a and Supplementary Figure5b. GAPDH was used as a loading control.

b) Confocal sections showing enhanced formation of Zyxin-positive positive FA in siOPTN transfected HUVECs plated 2hours on fibronectin in defined medium. Scale bar=10µm

c) Number and mean area of FA structures positive for Integrin alpha5 in siCtrl or siOPTN transfected HUVEC were quantified. Box plots show fold change to siCtrl condition, set to 1 (n=30 siCtrl cells and n=27 siOPTN cells, 3 independent experiments).Wilcoxon rank sum test (two-tailed):*,p=0.0286;**,p=0.0022

d) Representative maps of cell cortex elasticity measured in Figure5d. Control(siCtrl) or Optineurin knocked down(siOPTN) HUVEC cells, either non treated or treated with the Arp2-3 inhibitor CK666(siOPTN+CK666) are scanned with an AFM probe to determine Young's modulus in kPa(magnitude is color-coded from 0= purple to 30kPa=yellow). Scale bar=1mm.

e) Quantification using the imageJ software of the mean cell area in control(siCtrl) and Optineurin knocked down(siOPTN) HUVEC cells seeded on 5ug/mL Fibronectin-coated plates, either without(NT) or with the Rac inhibitor EHT1864 (Boxplots). One-way Anova with Tukey's test for multiple comparisons: ****, p≤0.0001. One representative experiment and 3technical replicates(n=2031 siCtrl cells, n=2606 siCtrl+EHT1864 cells, n=1850 siOPTN cells, n=2049 siOPTN+EHT1864 cells).

f) Adhesion of control or Flag-OPTN expressing HUVECs on 1.25, 2.5 and 5 µg/ml of fibronectin was assessed upon short-term plating of cells in 96well plates. Graphs represent the extent of adhesion to fibronectin as function of absorbance upon elution of the crystal violet dye. Bars represent means±SD of n2 independent experiments and 3 technical replicates(single dots). Two-way Anova with Sidak's test for multiple comparisons:*,p=0.0297,***,p =0.0003,****,p≤0.0001. The immunoblots show Flag-OPTN expression in transfected cells shown in Supplementary figure 5f and 5g. GAPDH was used as a loading control.

g

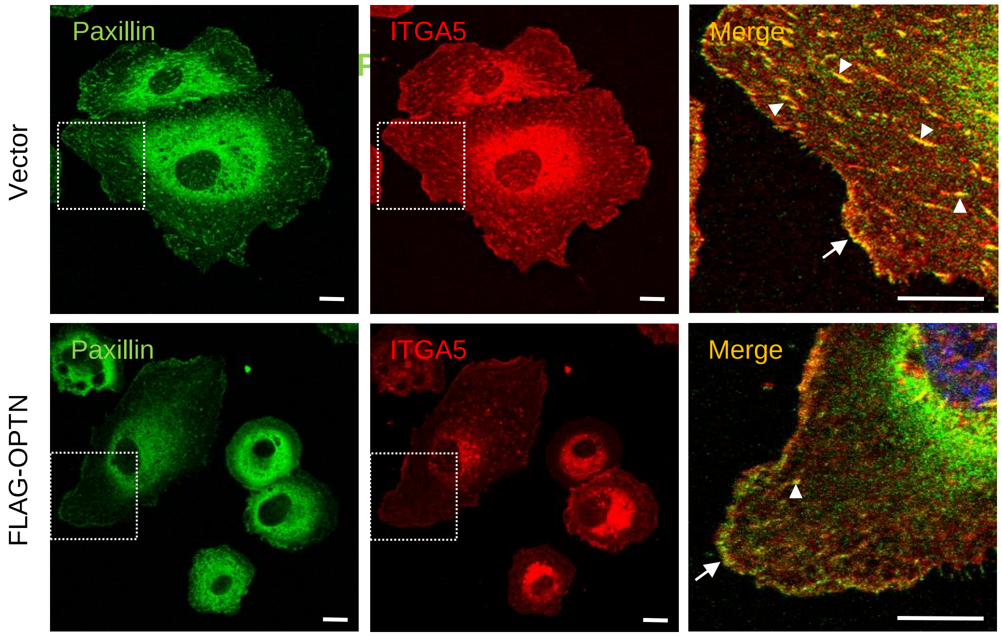

Supplementary FIGURE 5 g) Confocal sections showing reduced formation of integrin alpha-5-positive(ITGA5) and paxillin-positive Focal Adhesions(arrows heads) in control or Flag-OPTN expressing HUVECs. Arrows indicate peripheral Focal Complexes. Bar=10 mm. The square defines the region shown with merged labeling at x3.5 zoom. Representative experiment, n=3.

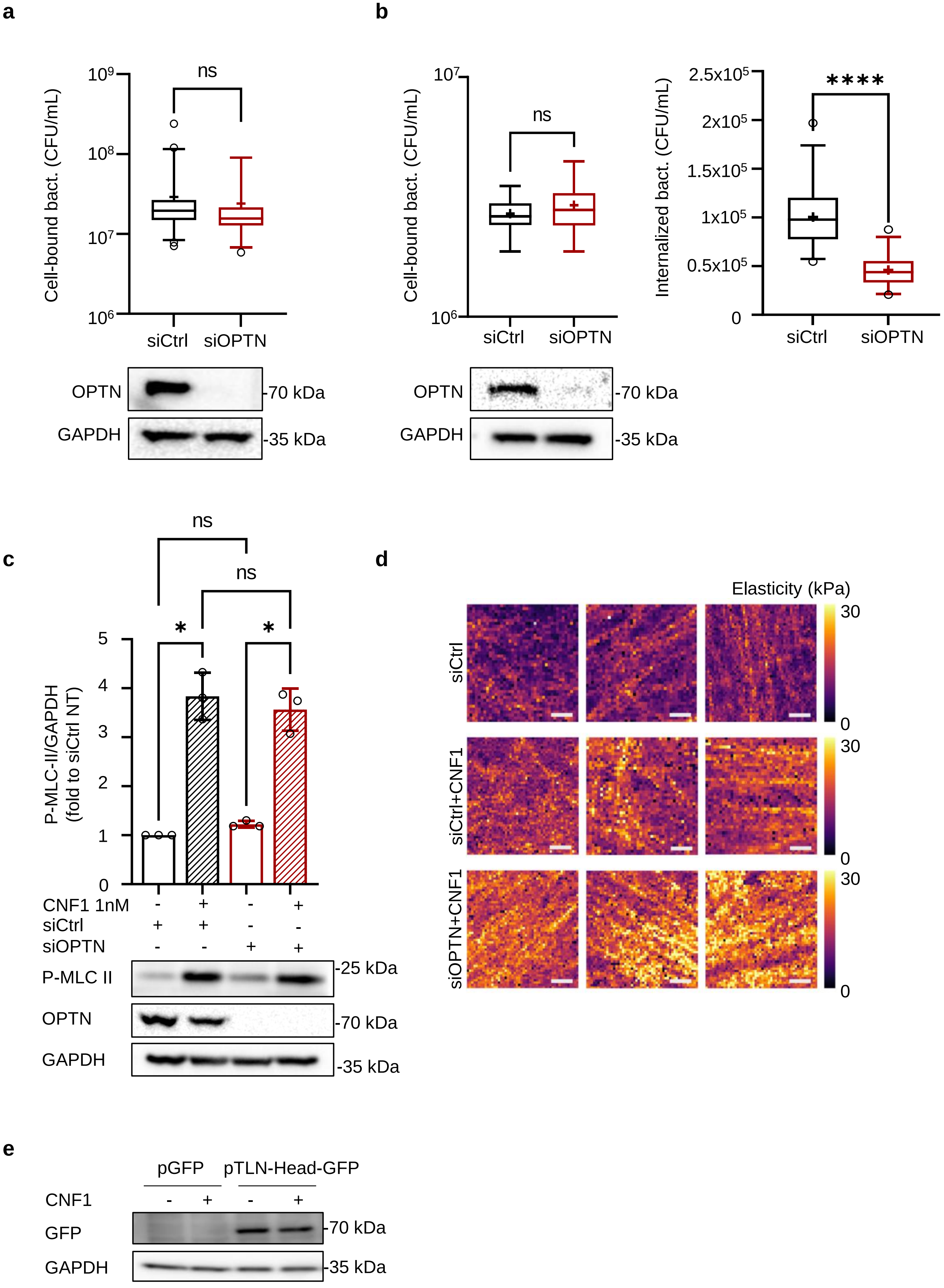

a
b
c
d
e
Cell-bound bact. (CFU/mL)
10^9
10^8
10^7
10^6
ns
siCtrl
siOPTN
OPTN
GAPDH
-70 kDa
-35 kDa
10^7
10^6
Internalized bact. (CFU/mL)
2.5x10^5
2x10^5
1.5x10^5
1x10^5
0.5x10^5
0
****
P-MLC-II/GAPDH
(fold to siCtrl NT)
5
4
3
2
1
0
*
CNF1 1nM
P-MLC II
-25 kDa
Elasticity (kPa)
30
0
siCtrl+CNF1
siOPTN+CNF1
pGFP
pTLN-Head-GFP
CNF1
GFP

Supplementary FIGURE 6

a)Top: Quantification of Ec bound to HUVEC cells transfected by siCtrl or siOPTN, MOI100. Boxplots represent absolute number of CFU/mL (Y axis starts at 10^6) of n=5independent experiments and at least 3 replicates per condition. Wilcoxon rank sum test(two-tailed):p=0.1314 not significant. **Bottom:**One representative immunoblot for verification of OPTN knock down. GAPDH is used as loading control.

b) Left: Quantification of Ec bound to cells in control (siCtrl) and Optineurin knocked down 5637 bladder epithelial cells (siOPTN) treated with 1nM CNF1. Boxplots display absolute number of CFU/ml (Y axis starts at 10^6) of n=3 independent experiments with at least 3replicates per condition,MOI100. Wilcoxon rank sum test(two-tailed):ns,p=0.5567 not significant. **Right:**Quantification of internalized Ec 30minutes after gentamicin treatment in control(siCtrl) or Optineurin knocked down 5637 bladder epithelial cells(siOPTN) treated with 1nM CNF1. Graph displays absolute CFU/mL. Box plots of n=3independent experiments with at least 3 replicates per condition. Wilcoxon rank sum test (two-tailed):****,p≤0.0001.**Bottom:**One representative immunoblot for verification of OPTN knock down in these experiments. GAPDH is used as loading control.

c)Quantification of Myosin light chain-II(P-MLC II) phosphorylation in control(siCtrl) or Optineurin knocked down(siOPTN) HUVEC cells, treated with CNF1 toxin at 1nM for 2hours. GAPDH has been used as a loading control, data show fold changes compared to non-treated siCtrl cells. Bars represent means±SD, n=3independent experiments.One-way Anova with Tukey's test for multiple comparisons:siCtrlNT vs siCtrlCNF1 *,p=0.0236; siOPTN NT vs siOPTN CNF1 *,p=0.0230; siCtrl CNF vs siOPTN CNF ns,p=0.3158; siCtrl NT vs siOPTN NT ns,p=0.0725, ns=not significant . Immunoblots below show a representative experiment, and the knock down verification.

d)Representative maps of cell cortex elasticity measured in Figure6c. Control(siCtrl) or Optineurin knocked down(siOPTN) HUVEC cells, either non treated or treated with 1 nM CNF1, are scanned with an AFM probe to determine Young's modulus in kPa(magnitude is color-coded from 0=purple to 30kPa=yellow). Scale bar=2µm.

e)Representative immunoblot showing transfection of the Talin-head plasmid fused to GFP compared to the empty vector(pGFP) in cells treated as in Figure6e.GAPDH is used as loading control.

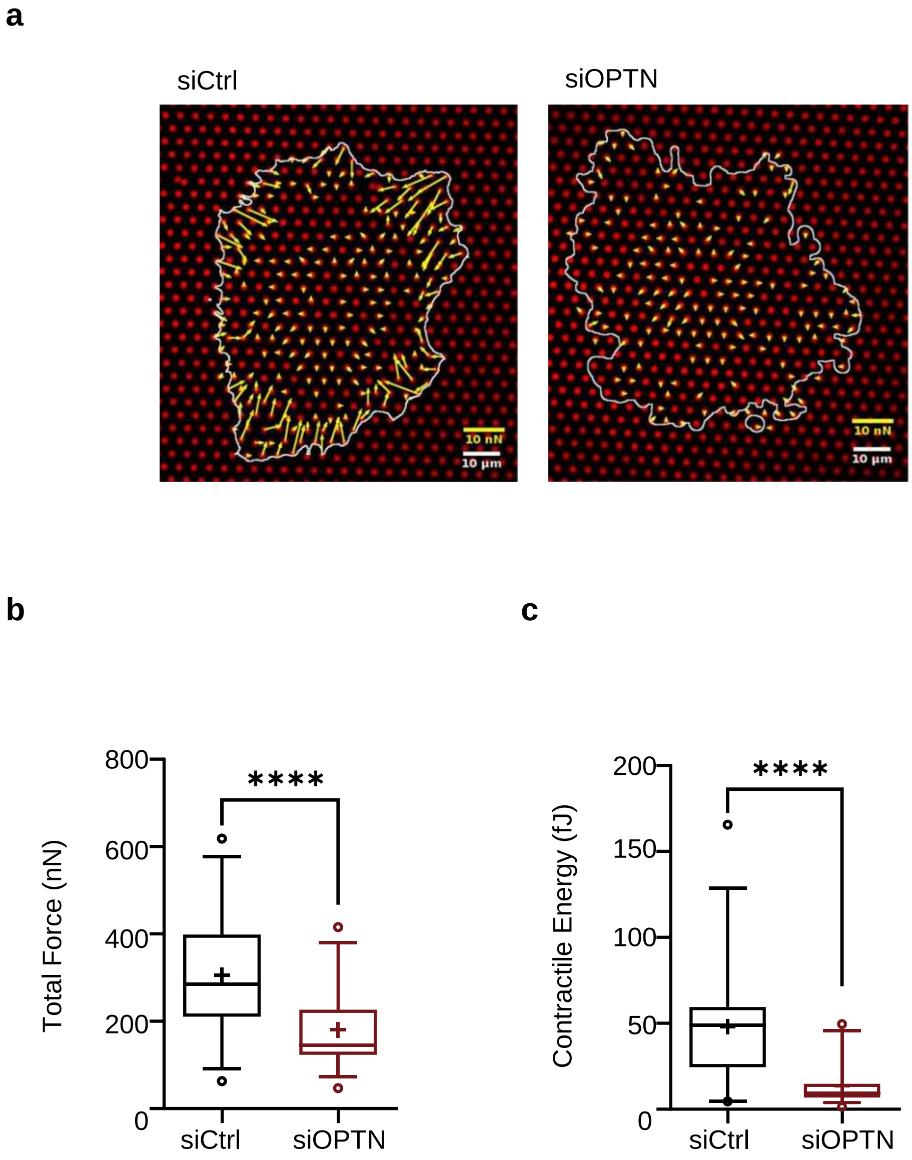
a
siCtrl
siOPTN
10 nN
10 µm
10 nN
10 µm
b
c
Total Force (nN)
800
600
400
200
0
****
siCtrl
siOPTN
Contractile Energy (fJ)
200
150
100
50
0
****
siCtrl
siOPTN

Supplementary FIGURE 7

a) Representative spatial distribution of traction force vectors (yellow arrows) in HUVEC transfected with siCtrl or siOPTN (outlined in white) and adhered 3h to micropillar substrates coated with fluorescent fibronectin (red). White scale bar, 10 µm. Yellow scale bar = 10 nN.

b) Total traction force exerted by a control (siCtrl) and Optineurin depleted (siOPTN) HUVEC cell represented in boxplots. Total force was calculated by adding the magnitude of all the force vectors in each cell. Forces are significantly lower for OPTN knocked down cells (n=32 siCtrl and n=33 siOPTN cells, 3 independent experiments, Wilcoxon rank sum test (two-tailed): ****, $p \leq 0.0001$).

c) Total contractile energy stored by a cell in the substrate. The total contractile energy was obtained by adding the strain energy of all the micropillars in each cell. Box plots of n=32 siCtrl and 33 siOPTN cells, 3 independent experiments, Wilcoxon rank sum test (two-tailed): p****≤0.0001).
