# Supplementary material for: Optineurin links Hace1-dependent Rac ubiquitylation to integrin-mediated mechanotransduction to control bacterial invasion and cell division
Source: Nat Commun. 2022 Oct 13;13:6059. doi: 10.1038/s41467-022-33803-x (PMC9561704; doi:10.1038/s41467-022-33803-x)
Supplement: Supplementary file 3 — Description of Additional Supplementary Files [file 41467_2022_33803_MOESM3_ESM.pdf]

Description of supplementary data 1- 4

Optineurin links Hace1-dependent Rac ubiquitylation to integrin-mediated mechanotransduction to control bacterial invasion and cell division

Serena Petracchini[1, #], Daniel Hamaoui[2, 3, #], Anne Doye[2, 3], Atef Asnacios[4], Florian Fage[4], Elisa Vitiello[5], Martial Balland[5], Sebastien Janel[6], Frank Lafont[6], Mukund Gupta[7], Benoit Ladoux[7], Jerôme Gilleron[8], Teresa M. Maia[9, 10, 11], Francis Impens[9, 10, 11], Laurent Gagnoux-Palacios[12], Mads Daugaard[13, 14], Poul H. Sorensen[15], Emmanuel Lemichez[1, 2, 3]* and Amel Mettouchi[1, 2, 3]*

[1] Institut Pasteur, Université Paris Cité, CNRS UMR6047, INSERM U 1306, Unité des Toxines Bactériennes, F-75015 Paris, France.

[2] Université Côte d'Azur, INSERM, C3M, Team Microbial toxins in host-pathogen interactions, Nice, France

[3] Equipe labellisée La Ligue contre le Cancer

[4] Université Paris Cité, CNRS, Laboratoire Matière et Systèmes Complexes, UMR7057, F-75013 Paris, France.

[5] Université Grenoble Alpes, CNRS, LiPhy, F-38000 Grenoble, France

[6] Université de Lille, CNRS, INSERM, CHU Lille, Institut Pasteur de Lille, U1019-UMR9017, CIIL- Center for Infection and Immunity of Lille, F-59000 Lille, France

[7] Université Paris Cité, CNRS, Institut Jacques Monod, F-75013 Paris, France

[8] Université Côte d'Azur, INSERM, C3M, Team Cellular and Molecular Pathophysiology of Obesity and Diabetes, Nice, France.

[9] VIB-UGent Center for Medical Biotechnology, VIB, Ghent, Belgium

[10] Department of Biomolecular Medicine, Ghent University, Ghent, Belgium.

[11] VIB Proteomics Core, VIB, Ghent, Belgium.

[12] Université Côte d'Azur, CNRS, INSERM, Institut de Biologie Valrose (iBV), Nice 06108, France

[13] Vancouver Prostate Centre, Vancouver, BC, V6H 3Z6, Canada.

[14] Department of Urologic Sciences, University of British Columbia, Vancouver, BC, Canada.

[15] Department of Molecular Oncology, BC Cancer Research Center, University of British Columbia, Vancouver, BC V5Z1L3, Canada.

[#] These authors contributed equally

* These authors jointly supervised this work

SUPPLEMENTARY DATA 1 and 2:

List of quantified proteins in HUVEC cells cultured on ECM of different stiffness (n=1,804) in the non-treated (NT, Supplementary data 1) or CNF1-treated condition (CNF, Supplementary data 2). Pairwise statistical tests (two-tailed moderated t-test for equality of means) were performed to reveal proteins that were significantly regulated under different substrate rigidity conditions. Columns from left to right contain: an indication whether the protein is significantly up- or downregulated, -log10 (adjusted p-value/FDR with Benjamini-Hochberg FDR control method for multiple hypothesis testing), log2 fold change ratio, Uniprot accession, protein name, gene name and identification score. Proteins are ordered by increasing FDR value for the 50kPa to 1kPa comparison test. Information in grey refers to protein groups that contain common proteomics experiments contaminant proteins.

SUPPLEMENTARY DATA 3.

List of proteins Z-scored LFQ intensities derived from the protein heatmap obtained after statistical testing and hierarchical clustering (n=1,076). Proteins are ordered according to heatmap on Figure2A. Pairwise statistical tests were performed to reveal proteins that were significantly regulated under different ECM rigidity conditions. Columns from left to right contain: Uniprot accession, protein name, gene name, cluster number and z-score per sample (empty cells represent undetected proteins). Information in grey refers to protein groups that contain common proteomics experiments contaminant proteins.

SUPPLEMENTARY DATA 4

Functional pathway enrichment analysis using DAVID. List of upregulated KEGG Pathways and Keywords for the Non treated (NT) and CNF1 (CNF) conditions. Table contains number of proteins per term (count column), P-value, list of genes and statistical tests (Bonferroni, Benjamini, FDR based on one-tail Fisher Exact test)
