# Supplementary material for: Optineurin links Hace1-dependent Rac ubiquitylation to integrin-mediated mechanotransduction to control bacterial invasion and cell division
Source: Nat Commun. 2022 Oct 13;13:6059. doi: 10.1038/s41467-022-33803-x (PMC9561704; doi:10.1038/s41467-022-33803-x)
Supplement: Supplementary file 5 — Reporting Summary [file 41467_2022_33803_MOESM5_ESM.pdf]

nature portfolio

Corresponding author(s): Mettouchi A., Lemichez E.

Last updated by author(s): Sep 12, 2022

Reporting Summary

Nature Portfolio wishes to improve the reproducibility of the work that we publish. This form provides structure for consistency and transparency in reporting. For further information on Nature Portfolio policies, see our Editorial Policies and the Editorial Policy Checklist.

Statistics

For all statistical analyses, confirm that the following items are present in the figure legend, table legend, main text, or Methods section.

n/a	Confirmed	
☐	☒	The exact sample size (*n*) for each experimental group/condition, given as a discrete number and unit of measurement
☐	☒	A statement on whether measurements were taken from distinct samples or whether the same sample was measured repeatedly
☐	☒	The statistical test(s) used AND whether they are one- or two-sided<br>*Only common tests should be described solely by name; describe more complex techniques in the Methods section.*
☐	☒	A description of all covariates tested
☐	☒	A description of any assumptions or corrections, such as tests of normality and adjustment for multiple comparisons
☐	☒	A full description of the statistical parameters including central tendency (e.g. means) or other basic estimates (e.g. regression coefficient) AND variation (e.g. standard deviation) or associated estimates of uncertainty (e.g. confidence intervals)
☐	☒	For null hypothesis testing, the test statistic (e.g. *F*, *t*, *r*) with confidence intervals, effect sizes, degrees of freedom and *P* value noted<br>*Give P values as exact values whenever suitable.*
☒	☐	For Bayesian analysis, information on the choice of priors and Markov chain Monte Carlo settings
☒	☐	For hierarchical and complex designs, identification of the appropriate level for tests and full reporting of outcomes
☒	☐	Estimates of effect sizes (e.g. Cohen's *d*, Pearson's *r*), indicating how they were calculated

Our web collection on statistics for biologists contains articles on many of the points above.

Software and code

Policy information about availability of computer code

Data collection	Western-blot chemiluminescnce was acquired on a FujiFilm LAS-3000 and a Syngene Pxi4 imaging system. Immunofluorescence images were acquired on on a Nikon A1R confocal microscope, with NIS-Elements software v 4.50.00. DNA content analysis was performed on a BD FACSAria flow cytometer.
Data analysis	Chemiluminescence images were quantified using ImageJ v2.3.1. Proteomics data analysis was performed with MacsQuant 1.6.11.0 and Limma package of R. Functional enrichment was analyzed in DAVID and network created in Cytoscape 3.9.1. The number and Mean area of focal adhesions were quantified using the image analysis software Motion Tracking 8.80.23(x64). Calculations were done in Excel when needed. TFM image processing and calculations were performed using MATLAB R2019a. AFM measurements were computed using Python 3.6.9 software. Data were tested for statistical significance with GraphPad Prism 9.0

For manuscripts utilizing custom algorithms or software that are central to the research but not yet described in published literature, software must be made available to editors and reviewers. We strongly encourage code deposition in a community repository (e.g. GitHub). See the Nature Portfolio guidelines for submitting code & software for further information.

Data

Policy information about availability of data

All manuscripts must include a data availability statement. This statement should provide the following information, where applicable:

- Accession codes, unique identifiers, or web links for publicly available datasets
- A description of any restrictions on data availability
- For clinical datasets or third party data, please ensure that the statement adheres to our policy

source data are provided as supplementary files. Materials can be obtained from the corresponding authors upon request. The mass spectrometry proteomics data have been deposited to the ProteomeXchange Consortium via the PRIDE partner repository with the dataset identifier PXD035396 (https://www.ebi.ac.uk/pride/).

Field-specific reporting

Please select the one below that is the best fit for your research. If you are not sure, read the appropriate sections before making your selection.

☒ Life sciences ☐ Behavioural & social sciences ☐ Ecological, evolutionary & environmental sciences

For a reference copy of the document with all sections, see nature.com/documents/nr-reporting-summary-flat.pdf

Life sciences study design

All studies must disclose on these points even when the disclosure is negative.

Sample size	No statistical methods were used to predetermine sample size before experimentation : sample sizes were based on previous published practices in the field, and in all cases were more than necessary to get statistically significant results. Proteomics experiment included 4 biological replicates/condition. Infection experiments included at least 3 biological replates in each condition. Adhesion assays included 3 replicates/condition. Cell area measure were done on more than 1000 cells on 3 replicates. Biophysics measurements were done on a total number of 17 to 45 cells depending on techniques as indicated for respective experiments. Bacteria growth was measured on 3 replicates.
Data exclusions	No data were excluded from the analysis
Replication	Experiments were repeated at least three times to be sure that the data shown in the paper is reproducible. Either the result of multiple experiments was used to calculate mean values, or representative experiments are shown, as indicated. All attempts from replications were successful.
Randomization	No randomization was done since experimental groups represented different genetic backgrounds (SiRNA knockdown)
Blinding	Imaging quantification was automated using Motion Tracking, hence manual counting by a blinded operator was avoided. CFU counting was done by an operator blinded to groups allocation.

Reporting for specific materials, systems and methods

We require information from authors about some types of materials, experimental systems and methods used in many studies. Here, indicate whether each material, system or method listed is relevant to your study. If you are not sure if a list item applies to your research, read the appropriate section before selecting a response.

Materials & experimental systems

n/a	Involved in the study	
☐	☒	Antibodies
☐	☒	Eukaryotic cell lines
☒	☐	Palaeontology and archaeology
☒	☐	Animals and other organisms
☒	☐	Human research participants
☒	☐	Clinical data
☒	☐	Dual use research of concern

Methods

n/a	Involved in the study	
☒	☐	ChIP-seq
☒	☐	Flow cytometry
☒	☐	MRI-based neuroimaging

Antibodies

Antibodies used	For western-blots:<br>mouse anti-HA clone 16B12 (HA.11, Covance MMS-101P) 1/2000<br>anti-c-myc clone 9E10 (11-667-149-001 Roche) 1/1000<br>anti-Flag M2 (Sigma, F3165) 1/1000<br>anti-Rac clone 102/Rac (BD Transduction Laboratories 610651) 1/1000,

anti-Flotilin clone 18/Flotillin-1 (BD Transduction Laboratories F65020) 1/5000,
anti-Transferrin Receptor clone H68.4 (Zymed 13-6800) 1/1000,
anti-Src clone L4A1 (Cell Signaling Technologies, CST 2108) 1/1000
anti-FAK clone 77/FAK (BD Transduction 610088) 1/1000
anti-OPTN (Abcam, 23666; Santa Cruz clone C-1, sc-271549) 1/500
anti-cyclin D1 clone M-20 (Santa Cruz sc-718) 1/500,
anti-p-FAK-Y397 clone 18/FAK(pY397) (BD Transduction 611807) 1/500,
anti-p-Src-Y416 clone D49G4 (CST 2101) 1/500,
anti-Paxillin clone 349 (BD Transduction 610052) 1/1000,
anti-p-Paxillin-Y118 (Invitrogen 44722G) 1/500,
anti p-P130CAS-Y410 (CST 4011) 1/500,
anti-p130CAS clone 35B.1A4 (Santa Cruz sc-20029) 1/1000, and
anti-GAPDH clone 0411 (Santa Cruz sc-47724) 1/5000,
anti-p-MLC Y18/S19 (Cell Signaling Technologies, CST 3674) 1/500.
Beta1 integrin clone 18/CD29 (BD 610468) 1/1000
ICAP-1 (ITGBP1) (Atlas Antibodies HPA071538) 1/500
Vinculin clone hVIN1 (Sigma V9131) 1/500
Talin 1/500 clone TA205 (Millipore 05-385)
HACE-1 (Custom-made, clone 9D3) 1/500
GFP clones 7.1 and 13.1 (Roche, 11814460001) 1/1000
For immunoaffinity precipitations: EZ view Red Anti-FLAG M2 Affinity Gel (Sigma, F2426) and EZ view Red Anti-c-Myc Affinity Gel (Sigma, E6654).
For immunofluorescence : paxillin clone 349 (BD-Transduction Laboratories 610052) 1/200, zyxin clone ZZ001 (Invitrogen 39-6000) 1/100, and integrin alpha 5 (AB1928, Millipore 1/400.

Validation

All commercial antibodies were used following manufacturer's instructions without further validation, except when blotting to assess RNAi efficiency which was also validating specificity of the primary antibody.
anti-Flotilin clone 18/Flotillin-1 (BD Transduction Laboratories F65020) validated for western blot analysis on Human, Mouse, Chicken.
anti-Transferrin Receptor clone H68.4 (Zymed 13-6800) validated for western blot and reactive on Human, Chicken, Hamster, Mouse, Rat
anti-Src clone L4A1 (Cell Signaling Technologies, CST 2108), reactive in Human Mouse Rat, suitable for Western blot
Anti-FAK clone77 (610088 BD bioscience) for Western blot, validated in human, rat, mouse, dog
anti-OPTN (Abcam 23666) Suitable for western blot validated for human and Santa Cruz clone C-1 sc-271549 is validated for western blot detection of Optineurin of mouse, rat and human origin. SiRNA verification provided also in this article (Santa Cruz C-1, Sup Fig5A, Sup Fig 6A-B and Abcam 23666, Fig 3B, Fig3D-E)
cyclin D1 Antibody (M-20) is validated for western blot detection of cyclin D1 of human origin
anti-p-FAK-Y397 clone 18/FAK(pY397) (BD Transduction 611807) is validated in human for western blot analysis
Phospho-Src Family (Tyr416) clone D49G4 (CST 2101) validated for western blot use in human mouse rat it detects Src only when phosphorylated at tyrosine 416
anti-Paxillin (BD Transduction 610052) verified for western blot and immunofluorescence use in human, reacts in human rat dog mouse
anti-p-Paxillin-Y118 (Invitrogen 44722G) validated in Western Blot, reacts in human and mouse
anti p-P130CAS-Y410 (CST 4011 Application in Western Blot, reactive in human, rat mouse
Anti-p130 Cas Antibody (35B.1A4) is recommended for detection of p130 Cas of mouse, rat and human origin by Western blot
Anti-GAPDH (0411) is recommended for GAPDH detection of human origin by western blot
anti-p-MLC Y18/S19 (Cell Signaling Technologies, CST 3674) is Recommended for western blot on human and mouse samples
Beta1 integrin clone 18/CD29 (BD 610468) is recommended for detection of Integrin β1 of human, mouse, rat, chicken and dog origin by western blot, SiRNA verification provided also in this article (supplementary Figure 1E) ICAP-1 (ITGBP1) (Atlas Antibodies HPA071538) verified for reactivity in human. RNAi validation of this antibody for western blot analysis is also provided in this article (Sup Fig 1G) Vinculin clone hVIN1 (Sigma V9131). Reactivity validated in bovine, canine, mouse, rat, turkey, human, chicken, frog. RNAi validation of this antibody for western blot analysis is also provided in this article (Sup Fig 1G) Talin TA205 (Millipore 05-385) validated in human rabbit mouse, RNAi validation of this antibody for western blot analysis is also provided in this article (Sup Fig 1G)
anti-human HACE-1 (Custom-made, clone 9D3) was validated by RNAi for western blot use (data not shown)
Anti-GFP clones 7.1 and 13.1 (Roche, 11814460001) is suitable to verify the expression of Green Fluorescent Protein (GFP) and GFP fusion pro¬teins by western blot analysis. Anti-Integrin α5 Antibody (AB1928, Chemicon) validated for immunofluorescence in human rat mouse chicken, anti-Zyxin clone ZZ001 (Invitrogen 39-6000) validated for immunofluorescence in human.

Eukaryotic cell lines

Policy information about cell lines

Cell line source(s)	HUVECs were pools from PROMOCELL . Bladder epithelial cell line 5637 are from ATCC (HTB-9)
Authentication	HUVEC cells are characterised by the vendor by FACs analysis of markers CD31, vWF, sm Actin<br>5637 cells are characterised by the vendor by karyotype verification and expression of AK-1, 1, ES-D, 1, G6PD, B, GLO-I, 1-2, Me-2, 1, PGM1, 1PGM3, 2
Mycoplasma contamination	HUVEC cells were tested negative for mycoplasma by the vendor and were used before passage 5.<br>5637 cells were tested negative for mycoplasma and used at passage Px+4
Commonly misidentified lines<br>(See ICLAC register)	no misidentified cell lines were used in this study
